# Biphasic regulation of CFTR expression by ENaC in epithelial cells: The involvement of Ca^2+^-modulated cAMP production

**DOI:** 10.3389/fcell.2022.781762

**Published:** 2022-08-30

**Authors:** Fulei Wuchu, Xiyang Ma, Yanting Que, Junjiang Chen, Ye Chun Ruan

**Affiliations:** ^1^ Department of Biomedical Engineering, Faculty of Engineering, The Hong Kong Polytechnic University, Kowloon, Hong Kong SAR, China; ^2^ Department of Physiology, Jinan University, Guangzhou, China; ^3^ Shenzhen Research Institute, Hong Kong Polytechnic University, Shenzhen, China

**Keywords:** the epithelial sodium channel (ENaC), cystic fibrosis transmembrane conductance regulator (CFTR), epithelial cells, endometrial epithelium, Ca2+, cAMP, sAC

## Abstract

The regulatory interaction between two typical epithelial ion channels, cystic fibrosis transmembrane conductance regulator (CFTR) and the epithelial sodium channel (ENaC), for epithelial homeostasis has been noted, although the underlying mechanisms remain unclear. Here, we report that in a human endometrial epithelial cell line (ISK), shRNA-based stable knockdown of ENaC produced a biphasic effect: a low (∼23%) degree of ENaC knockdown resulted in significant increases in CFTR mRNA and protein levels, CFTR-mediated Cl^−^ transport activity as well as intracellular cAMP concentration, while a higher degree (∼50%) of ENaC knockdown did not further increase but restored CFTR expression and cAMP levels. The basal intracellular Ca^2+^ level of ISK cells was lowered by ENaC knockdown or inhibition in a degree-dependent manner. BAPTA-AM, an intracellular Ca^2+^ chelator that lowers free Ca^2+^ concentration, elevated cAMP level and CFTR mRNA expression at a low (5 µM) but not a high (50 µM) dose, mimicking the biphasic effect of ENaC knockdown. Moreover, KH-7, a selective inhibitor of soluble adenylyl cyclase (sAC), abolished the CFTR upregulation induced by low-degree ENaC knockdown or Ca^2+^ chelation, suggesting the involvement of sAC-driven cAMP production in the positive regulation. A luciferase reporter to indicate CFTR transcription revealed that all tested degrees of ENaC knockdown/inhibition stimulated CFTR transcription in ISK cells, suggesting that the negative regulation on CFTR expression by the high-degree ENaC deficiency might occur at post-transcription stages. Additionally, similar biphasic effect of ENaC knockdown on CFTR expression was observed in a human bronchial epithelial cell line. Taken together, these results have revealed a previously unidentified biphasic regulatory role of ENaC in tuning CFTR expression involving Ca^2+^-modulated cAMP production, which may provide an efficient mechanism for dynamics and plasticity of the epithelial tissues in various physiological or pathological contexts.

## Introduction

Fluid (electrolytes and water) secretion and absorption across the epithelium are two major physiological functions of epithelial cells, which are tightly regulated for the homeostasis of epithelial cell-enriched organ systems such as respiratory, digestive, urinary and reproductive tracts. Two typical epithelial ion channels, cystic fibrosis transmembrane conductance regulator (CFTR) and the epithelial sodium channel (ENaC), are broadly expressed and responsible for Cl^−^ secretion (Anderson et al., 1991) and Na^+^ absorption (Palmer, 1992), respectively, driving water movement across the epithelium. The two channels are usually found in a reciprocal relationship (i.e. inversely related) (Berdiev et al., 2009), which is believed to maximize either epithelial secretion or absorption in the particular physiological context. For example, studies on the female reproductive system have shown that subject to hormonal changes over the estrus cycle in mice, uterine expression of CFTR is high at estrus phase (mid cycle in humans) and low at diestrus (late cycle in humans), while ENaC expression is, in the opposite way, low at estrus and high at diestrus (Chan et al., 2012). Such a cyclic expression pattern of these two channels is essential to the dominant uterine secretion to facilitate sperm transport at estrus, while uterine absorption to reduce luminal fluid and thus to stabilize embryo implantation at diestrus (Chan et al., 2009; Chen et al., 2012; Ruan et al., 2014).

The reciprocal relationship of CFTR and ENaC can result from hormonal regulation of their expression as observed in the female reproductive system (Ruan et al., 2014), although more studies have suggested that the two channels functionally inhibit each other (Chan et al., 2000; Konig et al., 2001), which could be through direct or indirect protein-protein interactions (Kunzelmann, 2001; Berdiev et al., 2007; Berdiev et al., 2009; Gentzsch et al., 2010). This is highlighted by the fact that Na^+^ absorption is abnormally elevated in defective airways in cystic fibrosis (CF) (Hobbs et al., 2013), a common genetic disease caused by CFTR mutations. Amiloride, a selective ENaC pharmaceutical blocker, was therefore once proposed to be used as a potential drug for CF. However, clinical trials have failed to show the effectiveness of amiloride or its more potent analogue, benzamil, for CF (Graham et al., 1993; Pons et al., 2000; Hirsh et al., 2004). Moreover, CFTR and ENaC were found to be dependent on each other rather than inhibiting in sweat gland (Reddy et al., 1999; Reddy and Quinton, 2003). It is therefore suggested that the molecular and functional regulatory mechanism between the two channels is rather complex and may be far from being fully understood.

Interestingly, for the recent decade, both CFTR and ENaC have been shown to play an additional role beyond epithelial secretion/absorption, which is to regulate a number of cellular signaling pathways involving cAMP, Ca^2+^, transcription factors, microRNAs and other signaling molecules for various physiological and pathological conditions (Chen et al., 2012; Lu et al., 2012; Ruan et al., 2012; Ruan et al., 2014; Sun et al., 2014; Dong et al., 2015; Fei et al., 2018; Madacsy et al., 2018; Sun et al., 2018a; Sun et al., 2018b). Moreover, the expression of these two channels has been shown subject to regulation by cAMP or Ca^2+^-dependent pathway (Dagenais et al., 2001; McCarthy and Harris, 2005; Dagenais et al., 2018). We therefore conducted the present study using endometrial epithelial cells as a model to understand if the modulation of ENaC would influence CFTR expression. To our surprise, the knockdown or inhibition of ENaC in endometrial epithelial cells resulted in a biphasic change of CFTR expression that is a low degree of ENaC deficiency upregulates CFTR, while further ENaC deficiency leads to downregulation of CFTR. We explored possible underlying mechanism and revealed the involvement of Ca^2+^-modulated cAMP production in such regulation of CFTR by ENaC.

## Results

### Knockdown of ENaC results in biphasic change of CFTR expression in endometrial epithelial cells

**FIGURE 1:**
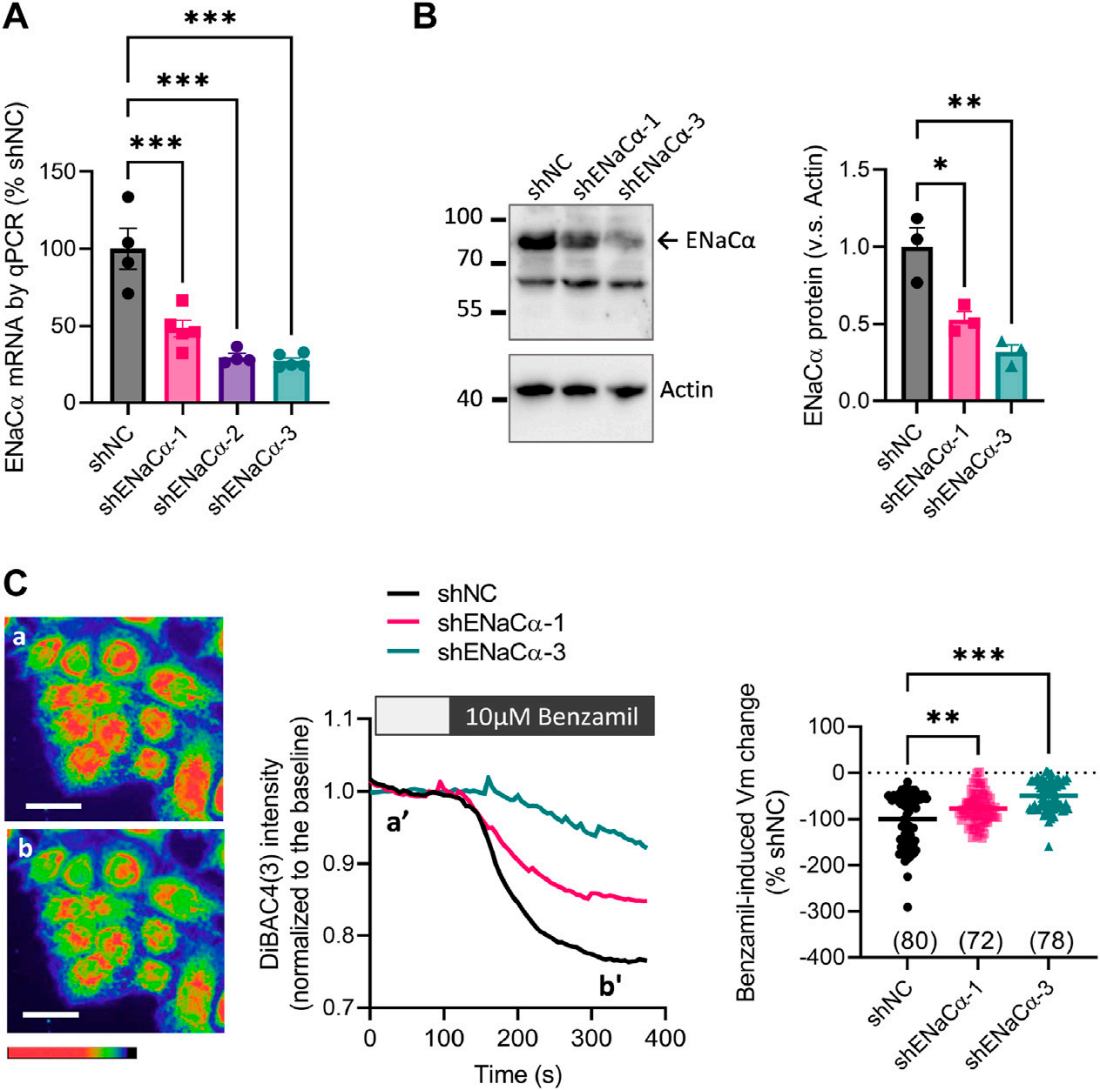
Different degrees of ENaC knockdown in ISK cells. **(A–B)** qPCR analysis of **(A)** and western blotting for **(B)** ENaCα in ISK cells with stable knockdown of ENaCα by transfection with lentivirus-packaged shRNAs against ENaCα (different designs: shENaCα-1, shENaCα-2 or shENaCα-3) or scrambled shRNAs as the negative control (shNC). **(C)** DiBAC4(3) measurement of membrane potential (Vm) with representative DiBAC4(3) fluorescent images (left), time-course traces of DiBAC4(3) intensity changes (middle), and quantifications (right) in the ISK lines treated with shNC, shENaC-1 or shENaC-3 before (a and a’) and after (b and b’) adding benzamil (10 μM), a selective inhibitor of ENaC, into the bath. Pseudo-colors in the images from red/yellow to blue/purple indicate Vm values from high to low. Scale bars = 50 µm. *N* = 4–5 **(A)**, 3 **(B)**, and 72-80 **(C)**. *p < 0.05, **p < 0.01, ***p < 0.001, one-way ANOVA with *Dunnett post hoc* test.

To specifically modify ENaC, we adopted shRNA-based knockdown of ENaCα, the rate limiting subunit of ENaC (Canessa et al., 1994), in Ishikawa (ISK) cells, a commonly used human endometrial epithelial cell line (Nishida, 2002; Sun et al., 2018b). Three designs of shRNAs, shENaCα-1, -2 and -3 with predicated knockdown efficiency of 64%, 78% and 95% respectively were packaged in lentivirus and used to stably knock down ENaC in ISK cells. As a result, the mRNA level of ENaCα was reduced to 48 ± 5%, 30 ± 2%, and 27 ± 2% in cells treated with shENaCα-1, -2 and -3, respectively, as compared to the line with scrambled shRNAs as the negative control (shNC) (Figure 1A). We next used the ISK lines with shNC, shENaCα-1 and shENaCα-3 (ISK-shNC, ISK-shENaCα-1 and ISK-shENaCα-3) for subsequent experiments. Western blot results confirmed that ENaCα protein level was knocked down by about 50% and 70% in ISK-shENaCα-1 and ISK-shENaCα-3 cells, respectively, as compared to that in ISK-shNC (Figure 1B). To confirm that ENaC was functionally knocked down, we examined ENaC channel function by measuring the membrane potential (Vm) with a voltage-sensitive fluorescent dye, DiBAC4(3), which we previously published (Ruan et al., 2012). Results showed that in all these ISK lines, the addition of benzamil (10 µM) induced a decrease in DiBAC4(3) intensity indicating Vm hyperpolarization (Figure 1C), consistent with the known role of active ENaC in endometrial epithelial cells in mediating Na^+^ influx and thus Vm depolarization. Such a benzamil-induced Vm hyperpolarization (indicating ENaC activity) was found to be reduced by about 23% and 50% in ISK-shENaCα-1 and ISK-shENaCα-3 lines, respectively, as compared to that in ISK-shNC (Figure 1C).

**FIGURE 2:**
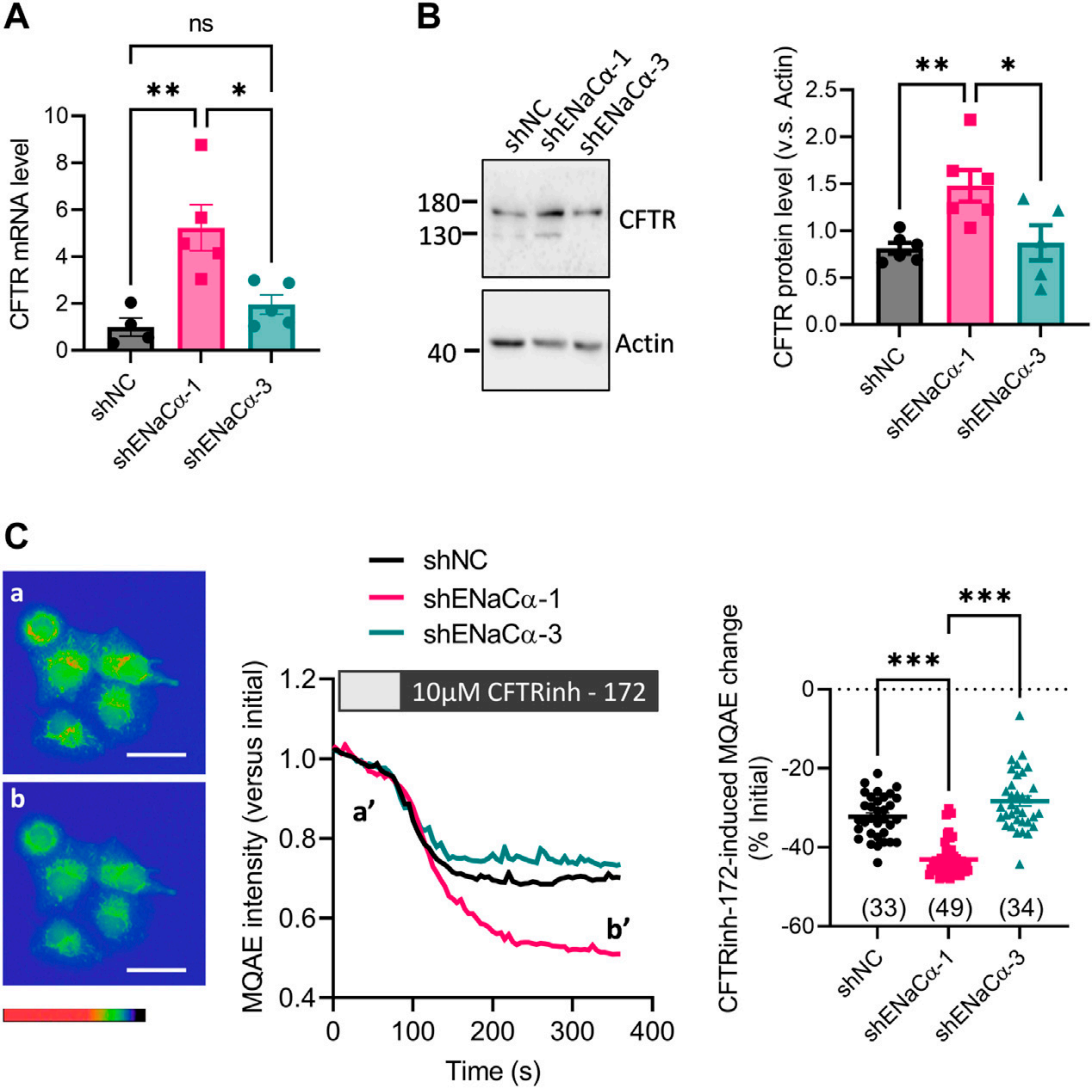
Effect of ENaC knockdown on CFTR expression in ISK cells. **(A–B)** qPCR **(A)** and representative western blot image with quantification **(B)** of CFTR in ISK cells treated with shNC, shENaC-1 or shENaC-3. **(C)** MAQE measurement of intracellular Cl^−^ concentration with representative MAQE fluorescent images (left), time-course traces (middle) and quantifications (right) in the ISK lines treated with shNC, shENaC-1 or shENaC-3 before (a and a’) and after (b and b’) the addition of CFTRinh-172 (10 µM), a selective inhibitor of CFTR. Pseudo-colors in the images from red/yellow to blue/purple indicate MAQE intensities (inversely correlated with Cl^-^concentrations) from high to low. Scale bars = 50 µm *N* = 4–5 **(A)**, 5-6 **(B)**, and 33–49 **(C)**. Ns: not significant. *p < 0.05, **p < 0.01, ***p < 0.001, one-way ANOVA with *Dunnett post hoc* test.

In these ISK lines with a low (ISK-shENaCα-1, 23%) or high (ISK-shENaCα-3, 50%) degree of ENaC knockdown, we measured the expression of CFTR. Quantitative PCR (qPCR) results showed that the CFTR mRNA level in ISK-shENaCα-1 significantly increased by about 4 folds as compared to that in ISK-shNC (Figure 2A). However, in ISK-shENaCα-3 where ENaC was more knocked down, the mRNA level of CFTR did not further increase but was significantly lower than that in ISK-shENaCα-1 (Figure 2A). Consistently, only ISK-shENaCα-1 but not ISK-shENaCα-3 showed higher CFTR protein level compared to that in ISK-shNC (Figure 2B). We next examined Cl^−^ channel function of CFTR in the ISK lines by measuring intracellular Cl^−^ levels using a Cl^−^-quenched fluorescent indicator, MQAE. While fluorescent intensity of MQAE was monitored under the microscope, a selective CFTR inhibitor, CFTRinh-172 (10 μM) was added onto the cells. Upon the addition of CFTRinh-172, there was a drop of fluorescent signal indicating an increase of intracellular Cl^−^ concentration and thus suggesting CFTR function in mediating Cl^−^ efflux in the cells (Figure 2C). Comparing these ISK lines, we found that ISK-shENaCα-1 exhibited a significantly larger CFTRinh-172-sensitive MQAE change compared to ISK-shNC (Figure 2C), indicating an increased gross CFTR activity in ISK-shENaCα-1. Whereas, in the ISK-shENaCα-3 with further ENaC knockdown, the CFTRinh172-sensitive change was similar compared to that in ISK-shNC, and significantly smaller than that in ISK-shENaCα-1 (Figure 2C). These results therefore consistently suggested that the regulation of CFTR by ENaC might be biphasic, i.e., CFTR upregulation by a low degree of ENaC deficiency but downregulation by further ENaC knockdown.

### Involvement of cAMP in the regulation of CFTR by ENaC

**FIGURE 3:**
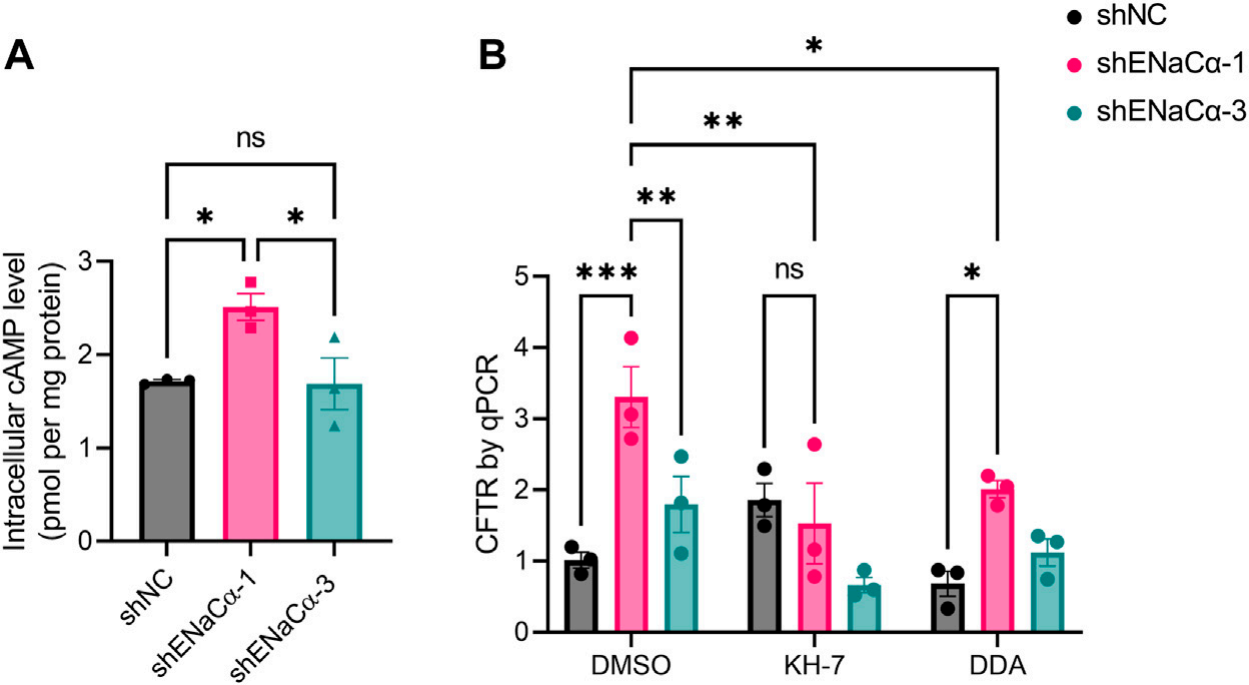
Involvement of cAMP in the regulation of CFTR expression by ENaC in ISK cells. **(A)** ELISA measurement of basal intracellular cAMP concentrations in the ISK lines treated with shNC, shENaC-1 or shENaC-3. Data were normalized to whole-cell protein levels. *N* = 3, ns: not significant. *p < 0.05, one-way ANOVA with *Dunnett post hoc* test. **(B)** qPCR analysis of CFTR mRNA levels in ISK lines treated with shNC, shENaC-1 or shENaC-3 after incubation with KH-7 (10 μM, an inhibitor of sAC), DDA (100μM, an inhibitor of mAC), or DMSO as the control for 24 h Ns: not significant. *p < 0.05, **p < 0.01, ***p < 0.001, two-way ANOVA with Tukey post hoc test.

We next asked how ENaC could regulate CFTR in such a biphasic manner. Since the promoter of CFTR gene is reported to contain CRE element that is activated by cAMP response element binding protein (CREB) (Cheng et al., 1991; McCarthy and Harris, 2005), we tested possible involvement of cAMP. We measured the intracellular cAMP concentration of the ISK lines, which interestingly showed that, without any treatment, ISK-shENaCα-1 had a significantly higher basal level of intracellular cAMP (2.51 ± 0.14 pmol/mg) as compared to that of ISK-shNC (1.71 ± 0.02 pmol/mg) (Figure 3A). ISK-shENaCα-3 had a significantly lower basal level of cAMP (1.69 ± 0.27 pmol/mg) than that in ISK-shENaCα-1, which was not different with that in ISK-shNC. Therefore, the effect of ENaC knockdown on basal cAMP level in ISK cells was also biphasic.

We next treated the cells with drugs that inhibit cAMP production, 5-dideoxyadenosine (DDA), a selective inhibitor of transmembrane adenylyl cyclase (tmAC), and KH-7, a selective inhibitor of soluble adenylyl cyclase (sAC). Interestingly, in the presence of KH-7 (10 µM, 24 h), the biphasic effect of ENaC on CFTR mRNA levels was found completely reversed with the CFTR mRNA increase in ISK-shENaCα-1 abolished (Figure 3B). However, although DDA (100 µM, 24 h) slightly lowered the CFTR mRNA level in all three ISK lines, the biphasic effect was not altered by DDA (Figure 3B). There results therefore suggested the involvement of sAC-mediated cAMP production in the regulation of CFTR by ENaC.

### Involvement of intracellular Ca^2+^ in the regulation of CFTR by ENaC

**FIGURE 4:**
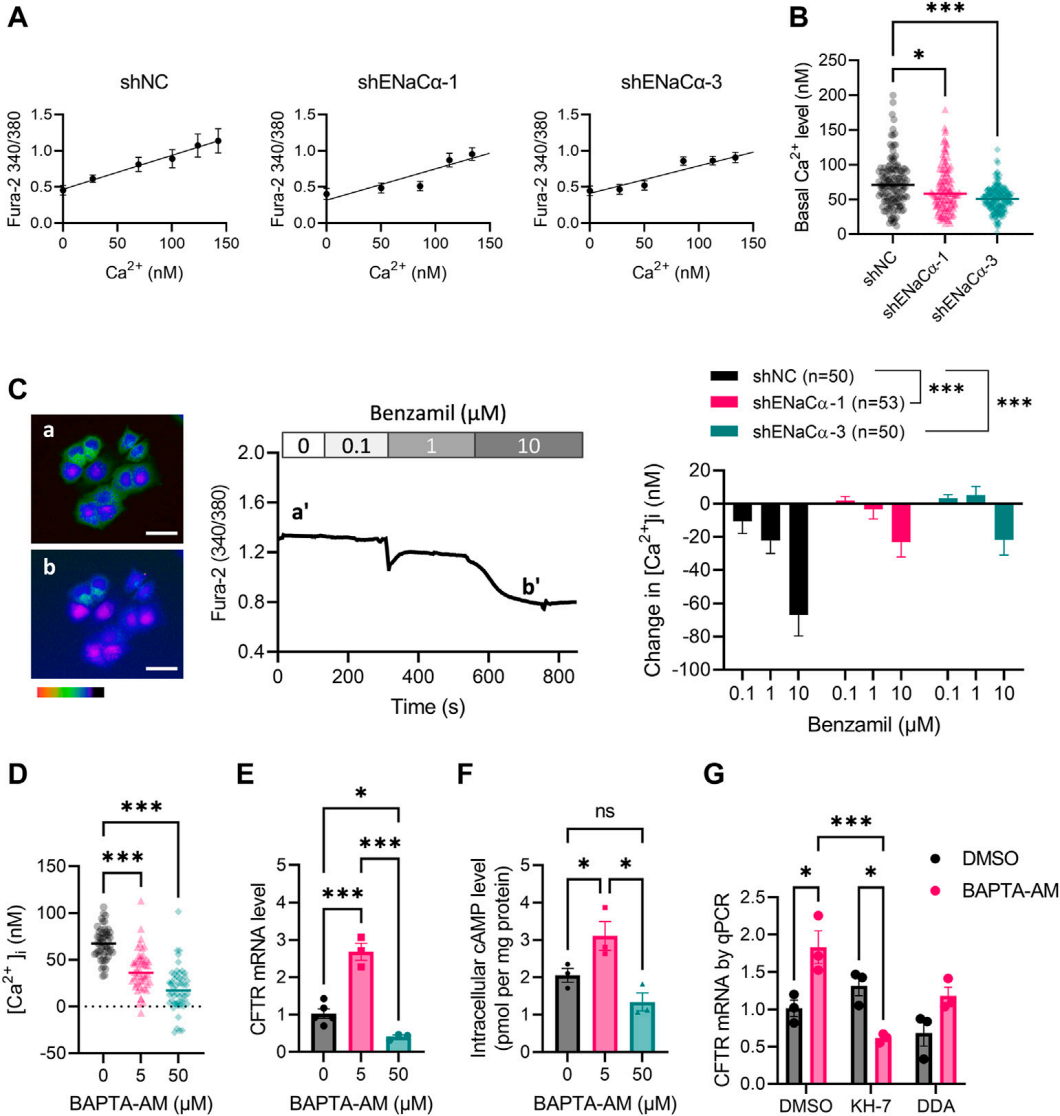
Involvement of intracellular Ca^2+^ in the regulation of CFTR expression by ENaC in ISK cells. **(A)** Intracellular Ca^2+^ concentrations ([Ca^2+^]_i_) of ISK lines with shNC, shENaC-1 or shENaC-3 were measured by Fura-2 imaging with calibration of Fura-2 intensity ratios at 340/380 nm into [Ca^2+^]_i_. **(B)** Basal [Ca^2+^]_i_ in the ISK lines were measured and calibrated accordingly. N = 137–153. *p < 0.05, ***p < 0.001, one-way ANOVA with *Dunnett post hoc* test. **(C)** Representative Fura-2 ratio images of ISK cells with time-course traces showing the change in [Ca^2+^]_i_ before (a and a’) and after (b and b’) the addition of benzamil (0.1–10 µM). Pseudo-colors in the images from red/yellow to blue/purple indicate Fura-2 340/380 ratios from high to low. Scale bars = 50 µm. Quantification of the benzamil-induced change in [Ca^2+^]_i_ in ISK lines with shNC, shENaC-1 or shENaC-3 is shown on the right. N is shown for each group. ***p < 0.001, two-way ANOVA. **(D)** [Ca^2+^]_i_ in ISK cells before (control) and after addition of BAPTA-AM (5–50 µM), an intracellular Ca^2+^ chelator. **(E)** qPCR analysis of CFTR mRNA levels in ISK cells treated with BAPTA-AM at 5 μM for 24 h or at 50 μM for 30 min. *N* = 3. *p < 0.05, ***p < 0.001, one-way ANOVA with *Dunnett post hoc* test. **(F)** ELISA measurement of intracellular cAMP in ISK cells after 1 h treatment with BAPTA-AM (5–50 μM) or DMSO as control in the presence of IBMX (100 μM). *N* = 3. Ns: not significant. *p < 0.05, one-way ANOVA with Newman-Keuls multiple comparisons test. **(G)** qPCR analysis of CFTR mRNA levels in ISK cells 24 h after treated with BAPTA-AM (5 μM), KH-7 (10 µM), DDA (100 µM) or DMSO. *N* = 3. *p < 0.05, ***p < 0.001, two-way ANOVA with *Tukey post hoc* test.

sAC activity is well noted to be modulated by intracellular Ca^2+^ level (Jaiswal and Conti, 2003). We previously observed in endometrial epithelial cells that ENaC activity affected intracellular Ca^2+^ level (Ruan et al., 2012). We therefore wondered if the regulation of CFTR by ENaC could possibly be through Ca^2+^. We measured intracellular Ca^2+^ levels ([Ca^2+^]_i_) in the ISK lines by Fura-2 imaging with calibration (Figure 4A), which showed that the basal [Ca^2+^]_i_ was significantly lower in ISK-shENaCα-1 (64.9 ± 2.8 nM) and ISK-shENaCα-3 (50.39 ± 1.9 nM), as compared to that in ISK-shNC (73.2 ± 3.0 nM) (Figure 4B). Consistently, the inhibition of ENaC by benzamil (0.1–10 µM) induced reduction in [Ca^2+^] a dose-dependent manner, although the response to benzamil was reduced when ENaC was knocked down in ISK-shENaCα-1 and ISK-shENaCα-3 (Figure 4C). To mimic a situation of intracellular Ca^2+^ reduction, we treated the cells with BAPTA-AM, an intracellular Ca^2+^chelator, which indeed produced decreases in [Ca^2+^]_i_ in ISK cells (Figure 4D). Interestingly, treating the cells with a low dose of BAPTA-AM (5 µM) for 24 h increased CFTR mRNA expression as compared to the DMSO control, while at a higher dose of 50 μM, it decreased mRNA CFTR expression in ISK cells (Figure 4E), mimicking the biphasic effect of ENaC knockdown. Similarly, intracellular cAMP level in ISK cells was found to be increased by the low dose (5 µM, 1 h) of BAPTA-AM treatment, but lowered by the higher dose (50 μM, 1 h), as compared to the control (Figure 4F). Moreover, the BAPTA-AM (5 µM)-induced increase in CFTR mRNA level was reversed by treatment with KH-7 (10 µM) and attenuated by DDA (10 µM) (Figure 4G). These results therefore suggested the intracellular Ca^2+^-modulated cAMP production underlying the regulation of CFTR by ENaC.

### Effect of ENaC deficiency on CFTR transcription in ISK cells

**FIGURE 5:**
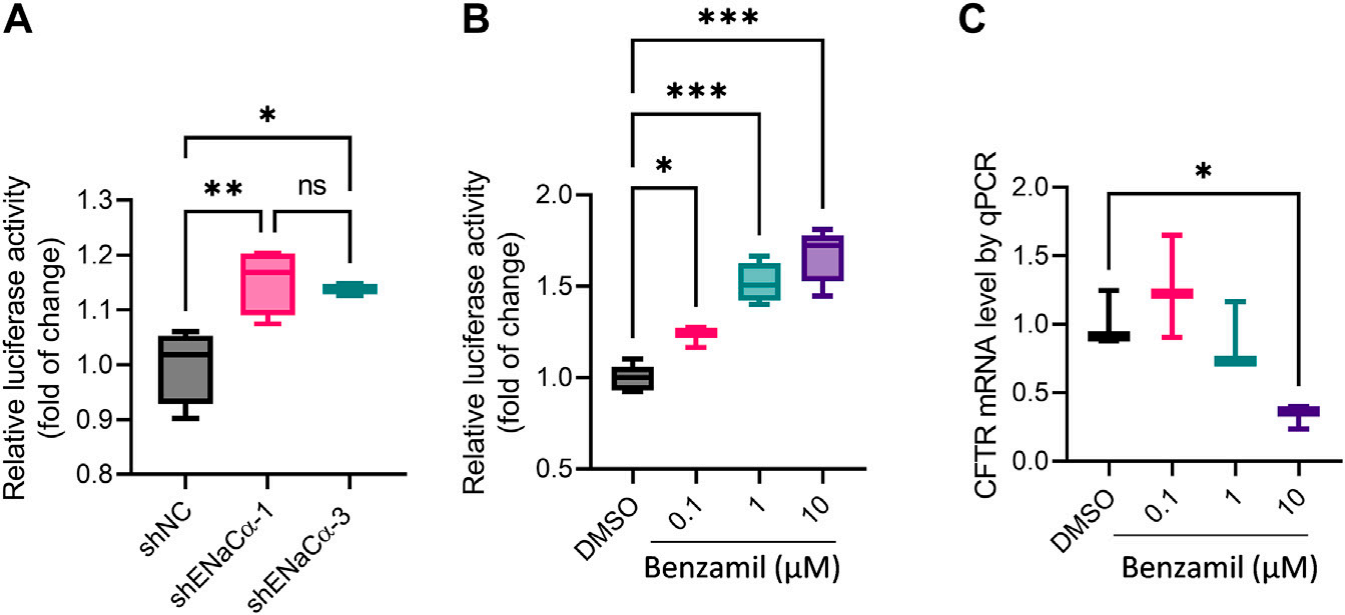
Effect of ENaC deficiency on CFTR transcription in ISK cells. **(A–B)** Measurement of CFTR transcription start by a dual luciferase reporter in ISK cells with shNC, shENaC-1 or shENaC-3 **(A)** or treated with benzamil (0.1–10 µM, 24 h, **(B)**. **(C)** qPCR analysis of CFTR mRNA levels in ISK cells treated with benzamil (0.1–10 µM, 24 h). *N* = 4 **(A)**, 3-6 **(B)** and 3 **(C)** *p < 0.05, ***p < 0.001, one-way ANOVA with *Dunnett post hoc* test.

To further understand the regulatory action of ENaC on CFTR expression, we adopted a dual luciferase reporter to indicate the initiation of CFTR transcription in cells (see method). To our surprise, the reporter activity was found significantly higher in both ISK-shENaCα-1 and ISK-shENaCα-3, as compared to that in ISK-shNC, with no difference between ISK-shENaCα-1 and ISK-shENaCα-3 (Figure 5A), suggesting that CFTR transcription was stimulated with both degrees of ENaC knockdown. We then treated ISK cells with benzamil to inhibit ENaC, and found that benzamil at all doses tested (0.1–10 μM, 24 h) enhanced the luciferase reporter activity in a dose-dependent manner (Figure 5B). However, we also measured CFTR mRNA in ISK cells by qPCR after the treatment with benzamil (0.1–10 μM, 24 h), which showed that at 0.1 µM benzamil slightly increased CFTR mRNA level, although at 10 μM, it significantly decreased CFTR mRNA (Figure 5C). These results may suggest that inhibition of ENaC may at one hand stimulate CFTR transcription, while at the other hand affect CFTR mRNA maturation or stability.

### Biphasic regulation of CFTR by ENaC in human bronchial epithelial cells

**FIGURE 6:**
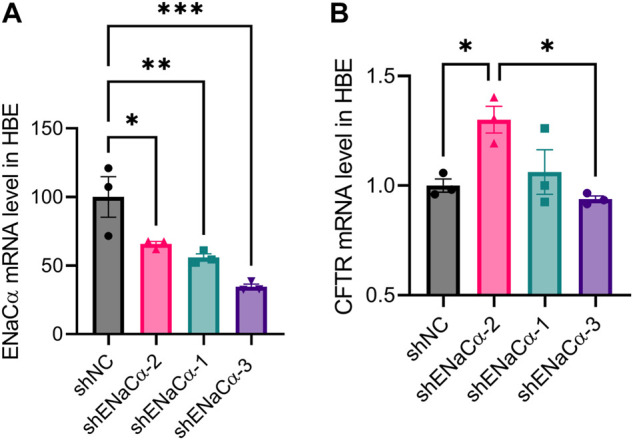
Effect of ENaC knockdown on CFTR expression in HBE cells. qPCR analysis of **(A)** ENaCα and **(B)** CFTR mRNA levels in HBE cells transfected with lentivirus-packaged shRNAs against ENaCα (three designs: shENaCα-1, shENaCα-2 and shENaCα-3). *N* = 3. *p < 0.05, ***p < 0.001, one-way ANOVA with *Dunnett post hoc* test.

Having observed the relationship between CFTR and ENaC, we wondered whether this phenomenon was specific to ISK cells. We established ENaC knockdown model in a human bronchial epithelial cell line (HBE). Stable transfection of shENaC-1, -2 and -3 resulted in ENaCα expression reduction in HBE cells by about 44%, 34% and 65% respectively (Figure 6A). Only with the lowest degree (34%) of ENaC knockdown (shENaCα-2) among the three, the mRNA expression of CFTR was found to be significantly increased compared to that in shNC-treated cells (Figure 6B). As ENaC was further knocked down (44%) by shENaCα-1, the mRNA level of CFTR started to drop, which significantly decreased when ENaC was knocked down to a high degree (65%) by shENaCα-3 (Figure 6B). Therefore, a similar biphasic regulation of CFTR by ENaC was also seen in HBE cells.

## Discussion

**FIGURE 7:**
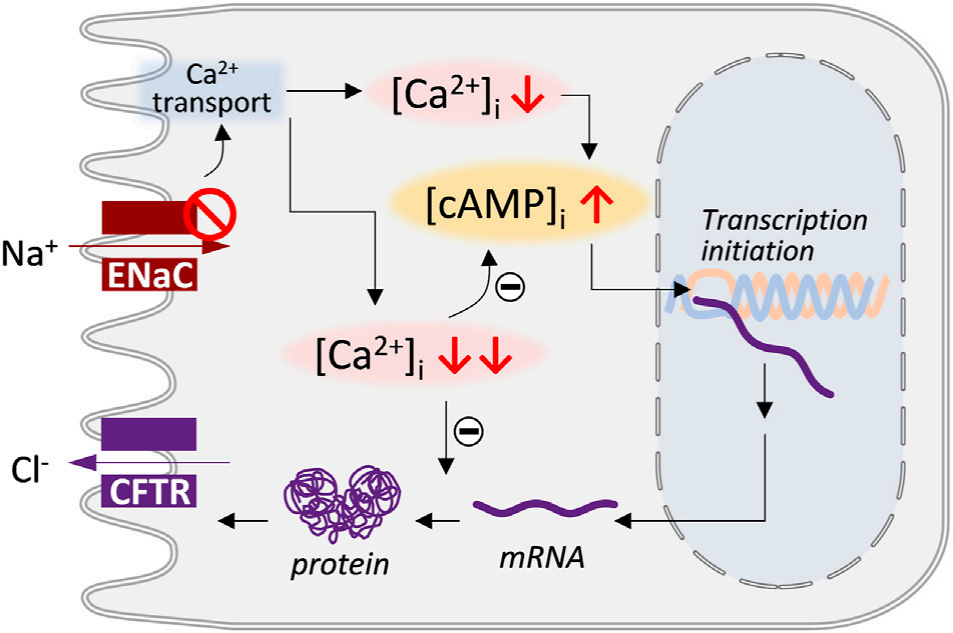
Schematic model showing biphasic regulatory action of ENaC on CFTR expression. Deficiency in ENaC, by affecting Ca^2+^ transport, may result in decrease in intracellular Ca^2+^ level ([Ca^2+^]_i_) which may increase intracellular cAMP level ([cAMP]_i_) and stimulate the transpiration start of CFTR gene. However, further inhibition of ENaC leading to massive drop in [Ca^2+^]_i_ may affect the stability of CFTR mRNA or protein resulting in CFTR downregulation.

In summary, the present study has demonstrated that CFTR functional expression in endometrial and airway epithelial cells may be subject to a biphasic regulation by ENaC (Figure 7). A low degree of ENaC deficiency may stimulate CFTR expression, while further attenuation of ENaC would not continue to increase but restore or even inhibit CFTR expression. Intracellular Ca^2+^-modulated sAC-driven cAMP production may be responsible for the positive regulation of CFTR by low-degree ENaC deficiency. The negative regulation on CFTR expression by the high-degree ENaC deficiency might occur at post-transcription stages.

The interaction between CFTR and ENaC as two ion channels key to epithelial homeostasis has been noted for long (Kunzelmann, 2001; Berdiev et al., 2007; Berdiev et al., 2009; Gentzsch et al., 2010). Using electrophysiological approaches, most of the previous studies recognized a reciprocal relationship between CFTR and ENaC channel open probability that when one is open the other tends to close or vice versa (Chan et al., 2000; Chan et al., 2001). Such a relationship is believed to be through quick protein-protein interactions (Kunzelmann, 2001; Berdiev et al., 2007; Gentzsch et al., 2010). Of note, gross ion channel activity is determined by both the open probability and plasma membrane density (or expression level) of the channel (Kashlan and Kleyman, 2011). The reciprocal relationship between the CFTR and ENaC was also observed at their expression levels, which was mostly seen in the female reproductive tract during menstrual cycle and believed to be a result of hormonal regulation (Chan et al., 2012). The present study has revealed that direct change in ENaC can affect CFTR gene transcription providing a mechanism how they interact at the expression level and in a relatively longer term. Moreover, such a regulatory effect on CFTR expression by ENaC is revealed to be not simply reciprocal but biphasic depending on the degree of ENaC deficiency. It therefore suggests the regulatory interaction between the two channels is far more complicated than originally thought, which may possibly explain why ENaC inhibitors failed to rescue CF airway (Graham et al., 1993; Pons et al., 2000; Hirsh et al., 2004), since a high degree of ENaC inhibition would not enhance CFTR but result in further CFTR deficiency. The degree of ENaC inhibition should be a key consideration for pharmaceutical design.

The present study also provides evidence that a cellular signaling pathway involving intracellular Ca^2+^, sAC, and cAMP may underly the biphasic regulation of CFTR expression by ENaC. First, inhibition or knockdown of ENaC caused reduction in [Ca^2+^]_i_, while lowing intracellular Ca^2+^ by BAPTA-AM to different extends produced similar biphasic effect of ENaC knockdown on CFTR mRNA and cAMP levels. Second, inhibiting sAC by KH-7 abolished the biphasic effect of ENaC knockdown or BAPTA-AM. Of note, Ca^2+^-activatable and inhibitable isoforms of mAC were identified to account for the dual role of Ca^2+^ in regulating cAMP levels (Antoni, 1997). However, the present data showed that mAC may not be essentially involved since the selective inhibition of mAC by DDA did not alter CFTR upregulation induced by ENaC knockdown or BAPTA-AM. Instead, sAC may be important here given the effect of KH-7 in abolishing ENaC knockdown or BAPTA-AM-induced CFTR upregulation. Although sAC has only been reported to be activated by Ca^2+^ (Jaiswal and Conti, 2003), dual role of Ca^2+^ in regulating cAMP levels was noted in sperm cells where sAC is well recognized to be essential to sperm cAMP production and thus sperm motility (Li et al., 2016). The present results suggest a possible inhibitory effect of Ca^2+^ on sAC in ISK cells too, which could be lifted by a low dose of BAPTA-AM or ENaC deficiency. The exact mechanism, however, awaits further investigation. Intriguingly, while the effects of ENaC knockdown on CFTR mRNA, protein and function levels were biphasic depending on the knockdown degree, the luciferase reporter assay showed that all tested degrees of ENaC inhibition or knockdown stimulated CFTR transcription, suggesting that the negative regulation on CFTR expression by the high-degree ENaC deficiency might occur at post-transcription stages. It has been shown that Ca^2+^ participates in various signaling pathways that determine mRNA stability and thus protein expression (Misquitta et al., 2006). Possibly, massive reduction in Ca^2+^ as a result of high-degree ENaC deficiency reduced CFTR mRNA or protein stability in ISK cells (Figure 7). This may suggest that the biphasic relationship between ENaC and CFTR is a combined effect of transcription initiation and mRNA or protein stability reduction. The mechanisms underlying such a complex biphasic regulatory relationship will need to be further explored. Attention should be paid to the regulatory action of CFTR onto ENaC as well, given the recognized roles of CFTR in regulating multiple signaling pathways (Chen et al., 2012; Lu et al., 2012; Ruan et al., 2014; Dong et al., 2015; Sun et al., 2018a).

It should be noted that CFTR is presently shown to be only upregulated within a very small window (about 20%–30% ENaC deficiency) in the endometrial or bronchial epithelial cell models, suggesting a tight and complex regulation between ENaC and CFTR. Such a regulatory relationship should be of physiologically significant. In the endometrial epithelium, for an example, which undergoes dynamic changes and cyclic remodeling during the menstruation cycle, where ENaC and CFTR expressions are known to fluctuate (Ruan et al., 2014), the regulation between epithelial ion channels may provide an efficient mechanism for epithelial plasticity and self-remodeling. In other epithelial tissues such as the airway where the epithelial cells are subject to many environmental dynamics (e.g., airway pathogens), the tight regulation to realize the switch between secretion and absorption or other epithelial cell activities might also be of physiological or pathophysiological importance.

## Materials and methods

### Cell culture

Human endometrial epithelial cell line Ishikawa (ISK) was purchased from ATCC (Virginia, United States) and cultured in RPMI-1640 (Thermo Fisher, 31800022) supplemented with 10% fetal bovine serum (v/v) and 1% penicillin–streptomycin (v/v) in 5% CO_2_ incubators at 37°C. The cell line was authenticated by STR profiling at the Department of Anatomical and Cellular Pathology, Faculty of Medicine, The Chinese University of Hong Kong. Human bronchial epithelial cell line (16HBE14o-, ATCC) was a gift from Prof Zhou Wenliang at Sun Yat-sen University and cultured in Minimal Essential Medium (Thermo Fisher, 41500034) supplemented with 10% fetal bovine serum (v/v) and 1% penicillin–streptomycin (v/v) in 5% CO_2_ incubators at 37°C.

### ENaC knockdown

Three designs of shRNAs targeting ENaCα and the scrambled non-coding shRNAs (shNC) were purchased from (Sigma-Aldrich) (Table 1). To package the shRNAs into the lentivirus, envelope vector pMD2.G (Addgene, 12259), packaging vector psPAX2 (Addgene, 12260) and shRNAs together with lipofectamine 2000 (ThermoFisher Scientific) were transfected into 293FT cells. Packaged lentivirus was harvested 72 h after transfection and next transfected into ISK or HBE cells for 24 h in the presence of polybrene (6 μg/ml). The cells were then cultured in the presence of puromycin (1 μg/ml) for 14 days to achieve stable knockdown.

**TABLE 1:** ShRNA information.

	TRC no.	Sequence (from 5’ to 3’)	Predicted knockdown efficiency
shENaCα-1	TRCN0000044558	CCG​GCC​AGA​ACA​AAT​CGG​ACT​GCT​TCT​CGG​AAG​CAG​TCC​GAT​TTG​TTC​TGG​TTT​TTG	64
shENaCα-2	TRCN0000044562	CCG​GCG​CAG​AGC​AGA​ATG​ACT​TCA​TCT​CGA​GAT​GAA​GTC​ATT​CTG​CTC​TGC​GTT​TTT​G	78
shENaCα-3	TRCN0000044559	CCG​GCG​ATG​TAT​GGA​AAC​TGC​TAT​ACT​CGA​GTA​TAG​CAG​TTT​CCA​TAC​ATC​GTT​TTT​G	95

### Quantitative PCR

Cells were lysed in TRIzol (Thermo Fisher Scientific, 15596026) for RNA extraction according to the manufacturer’s instructions. 1 μg RNA was then reverse transcribed into cDNA using High-Capacity cDNA Reverse Transcription kit (Thermo Fisher Scientific, 4368814) following the manufacturer’s instructions. Afterward, targeting genes (Table 2, all sequences were listed from 5′ to 3′) were amplified and detected by Touch Real-Time PCR Detection System (Bio-Rad, CFX96) using STBR Green Master mix (TAKARA, RR420A). GAPDH was used as a housekeeping gene for normalization. The data were analyzed using ΔΔCT method.

**TABLE 2:** Primer list.

Gene	Forward	Reverse
Human ENaCα	AGC​TCC​TCC​AGC​TCC​TCT​TT	CAG​CCT​CAA​CAT​CAA​CCT​CA
Human CFTR	GTG​TGA​TTC​CAC​CTT​CTC​CAA	GCC​TGG​CAC​CAT​TAA​AGA​AA
Human GAPDH	AGGGTCATCATCTCTGCC	CCATCACGCCACAGTTTC

### Membrane potential imaging

Cells were seeded on coverslips and grown for 24 to 48 h before the experiment. Cells on the coverslip were washed off the growth medium with Margo-Ringer buffer (NaCl 130 mM, KCl 5 mM, MgCl_2_ 1 mM, CaCl_2_ 2.5 mM, Hepes 20 mM and glucose 10 mM, pH 7.4), mounted to a chamber containing 1 ml Margo-Ringer buffer with the presence of a membrane potential-sensitive fluorescence dye, DiBAC4(3) (1 μM, Thermo Fisher Scientific, B438) and then placed under a fluorescence microscope (Nikon, ECLIPSE Ti2). After 15 min stabilization, the DiBAC4(3) was excited at 488 nm with emission lights collected at 535 nm and pictures taken every 3–5 sec. The data were collected and analyzed through NIS-Elements software.

### Western blot

Cells were lysed in pre-cooled RIPA lysis buffer (50 mM Tris-Cl, pH 7.5, 150 mM NaCl, 1% NP-40, 0.5% DOC and 0.1% SDS) with protease inhibitor cocktail (Thermo Fisher Scientific, 78443) for 30 min on ice. The supernatant was collected after centrifugation at 13,000 rpm for 30 min at 4°C. The protein concentration of each sample was measured with a BCA protein assay kit (Beyotime, P0011). Proteins were denatured in LDS sample buffer (Thermo Fisher Scientific, NP0007) plus 4% β-mercaptoethanol (Acros, 125472500) with heating at 70°C for 10 min to detect ENaCα, or incubated at room temperature for 30 min to detect CFTR. After denaturation, proteins were separated by SDS-polyacrylamide gel electrophoresis and subsequently transferred to equilibrated nitrocellulose membrane. The membrane was blocked by 5% non-fat milk in TBS for 30 min and subsequently incubated with the primary antibody in 5% non-fat milk in TBS at 4°C overnight. Primary antibodies against CFTR (1:500, Alomone Labs, ACL-006), ENaCα (1:500, StressMarq, SPC403), and actin (1:2000, Sigma Aldrich, MAB1501R) were used. After three washes with 0.05% Tween 20 in TBS, the membrane was incubated with HRP-conjugated antibodies at room temperature for 1 h and visualized by ECL substrates (Bio-Rad, 170-5060) and ChemiDoc MP Imaging System (Bio-Rad). HRP-conjugated secondary antibodies were goat anti-rabbit IgG (1:2,000, Bio-Rad, 12004159) and goat anti-mouse IgG (1:2,000, Bio-Rad, 12004159). Image J software was used for the densitometry of western blots.

### Intracellular Cl^-^ imaging

Cells were seeded on coverslips and grown for 24 to 48 h before the experiment. Prior to imaging, cells on the coverslip were incubated with a Cl^-^ sensitive fluorescent dye, MQAE (10 mM, MedChemExpress, HY-D0090) in Margo-Ringer buffer for 30 min at 37°C followed by washing and stabilizing for another 10 min. The coverslip was then mounted to a chamber containing 1 ml Margo-Ringer buffer under a fluorescence microscope (Nikon, ECLIPSE Ti2). MQAE was excited at 340 nm with emission at 460 nm every 3–5 sec. The data were collected and analyzed through NIS-Elements software.

### Intracellular cAMP measurement

Intracellular cAMP concentration was measured with Direct cAMP ELISA Kit (Enzo Life Sciences, Cat. No. ADI-900-066). Cells were lysed with 0.1M HCl at room temperature for 10 min. The lysates were centrifuged at 1000 g for 5 min, and the supernatants were used for subsequent analysis. Part of the supernatants were used to determine protein concentration in each sample with BCA Protein Assay Kit (Beyotime, Cat No. P0011). Another part of the supernatants was used for cAMP measurement, added with acetylating reagent (10 μl for every 200 μl sample) to increase assay sensitivity. Sample wells, cAMP standard wells and other reference wells were loaded and treated according to assay layout sheet and product manual. Optical density (OD) of each well was read at 405 nm with Ledetect 96 Absorbance Plate Reader (Labexim Products), subtracting mean OD of substrate blank from all measurements. Concentration of cAMP of each sample was calculated with a four-parameter logistic curve fitting program.

### Ca^2+^ imaging and calibration

Cells seeded on coverslips were incubated with Fura-2 (2 μM Thermo Fisher Scientific, F1221) for 30 min at 37°C before mounted onto a fluorescence microscope (Eclipse Ti, Nikon, Tokyo, and Japan). Fura-2 fluorescence was alternately excited at 340 and 380nm, and the emission signals were recorded at 510 nm. Ca^2+^ calibration was done according to a previous protocol (Williams and Fay, 1990). Briefly, cells were washed with Ca^2+^-free bath solution containing (in mM): NaCl 130, KCl 5, Hepes 20, EGTA 10 and glucose 10 (pH 7.2) before loaded with Fura-2 (2 μM) in the bath solution at 37°C for 30 min. The coverslip was then transferred to a mini chamber containing 1 ml Ca^2+^-free bath solution supplemented with nigericin (5 μM) and ionomycin (2 μM), and mounted on to the fluorescence microscope. To calibrate the change in Fura-2 fluorescence intensity into Ca^2+^ concentrations, a series of volumes of a high Ca^2+^ buffer containing (in mM): NaCl 130, KCl 5, CaCl_2_ 10, Hepes 20, EGTA 10 and glucose 10 (pH 7.2) supplemented with nigericin (5 μM) and ionomycin (2 μM) was added into the Ca^2+^-free bath to achieve CaEGTA concentrations at 0, 1, 2, 4, 6, 8, and 10 mM. The concentration of free Ca^2+^ was estimated using the equation: [Ca^2+^]_free_ = K_d-EGTA_ x (C_CaEGTA_/C_K2EGTA_), where K_d-EGTA_ is a constant of 150.5 at 20°C with a pH of 7.2. The calibration curve was obtained by fitting Fura-2 240/380 ratios with [Ca^2+^]_free._

### Luciferase activity assay

The proximal 5′region of human *CFTR* (- 1930 to +70, where +1 represents the transcriptional start site of *CFTR*) was amplified and cloned into pGL3-basic luciferase reporter by *KpnI* and *NheI* sites to make the pGL3/hCFTR construct (MiaoLingPlasmid Company). ISK cells were seeded into 96-well culture plates at a density of 1 × 10^4^ cells/well and co-transfected with luciferase reporter plasmid (pGL3/hCFTR, 1,000 ng/well) and internal control plasmid (Renilla-Luc, 10 ng/well) using Lipofectamine 2000 (Invitrogen). Firefly and renilla luciferase activities were assessed using the Dual-Glo Luciferase Assay System (Promega, E2920) according to the manufacturer’s instructions. The relative luciferase activity was defined as the ratio of readout for firefly luciferase to that for renilla luciferase with that of control group set as 1.0. Triplicate wells were used for measurement at each group.

### Statistics

One-way ANOVA was used for comparisons among more than 2 groups. Two-way ANOVA was used when there were two different categorical independent variables. A *p* value smaller than 0.05 was regarded as statistically significant. All graphs were generated and all statistical analyses were done with GraphPad Prism 9.

## Data Availability

The original contributions presented in the study are included in the article/supplementary material, further inquiries can be directed to the corresponding author.
